# Morbidity and Mortality Profile of Leishmaniasis in an Andean Region of Ecuador in the Context of Climate Change

**DOI:** 10.3390/tropicalmed10090254

**Published:** 2025-09-03

**Authors:** Enma Veronica Páez-Espinosa, Delia Maria Sosa-Guzmán, Luis René Buitrón-Andrade, Nicole Dávila-Jumbo, Martín Israel Cáceres-Ruiz, Vinicio Francisco Robalino-Flores, Eugènia Mato-Matute

**Affiliations:** 1 Department of Clinical Laboratory, School of Medicine, Pontifical Catholic University of Ecuador, Quito 170143, Ecuador; dmsosag@puce.edu.ec (D.M.S.-G.); davilajnico30@gmail.com (N.D.-J.); martincr2000@gmail.com (M.I.C.-R.); vrobalino671@puce.edu.ec (V.F.R.-F.); 2 Center for Research on Health in Latin America (CISeAL) San Pedro, Quito 170170, Ecuador; 3 Faculty of Health and Wellness, School of Medicine, Pontifical Catholic University of Ecuador, Quito 170143, Ecuador; rbuitron@puce.edu.ec; 4 Department of Endocrinology and Nutrition, Hospital de la Santa Creu i Sant Pau, 08041 Barcelona, Spain; emato@santpau.cat; 5 Networking Research Centre of Bioengineering, Biomaterials and Nanomedicine (CIBER-BBN), C/Monforte de Lemos 3-5, 28029 Madrid, Spain

**Keywords:** leishmaniasis, climate change, Andean slope, morbidity, Ecuador

## Abstract

Leishmaniasis is a parasitic disease transmitted by female sandflies of the genus Lutzomyia. Ecuador is divided into three distinct natural regions: the Andes, the Coast, and Amazonia, each characterized by significant variations in altitude and climate. While most reported cases of leishmaniasis are associated with humid, low-altitude rural areas, our study uncovered an unexpected trend: confirmed cases occurring in traditionally cold Andean regions. To investigate this issue, we conducted a cross-sectional ecological study using official morbidity and mortality records from the Ecuadorian Health Care Registration Platform, focusing on the cantons of Alausí and Chunchi in Chimborazo Province from 2013 to 2022. Chimborazo Province, in the Andes, is characterized by higher altitudes (2740 m above sea level) and a cold climate (averaging 13 °C throughout the year). Among a population of 44,089 residents in Alausí, we reported a total of 40 confirmed cases, with 97.5% classified as cutaneous and 2.5% as mucocutaneous, predominantly affecting children and males. No mortality cases were recorded during the study period. To further explore environmental influences, we examined the Alausí region, where climate change has led to rising average temperatures, deforestation, and changes in humidity levels. Leishmaniasis cases in Alausí showed seasonal peaks, particularly in 2018 and 2019, correlating with warmer and more humid conditions. Environmental factors such as temperature and humidity were strongly associated with the prevalence of the disease, suggesting that climate change may be increasing transmission risks. These findings point to the value of incorporating environmental monitoring into public health strategies for vector-borne diseases that affect vulnerable populations in the Andes.

## 1. Introduction

Leishmaniasis is a persistent, vector-borne zoonotic infection caused by protozoan parasites from the genus *Leishmania* [1]. The bite of infected female Phlebotomine sand flies transmits the *Leishmania flagellates* to vertebrates. These flies are hosted by canids, rodents, marsupials, mongooses, bats, and hyraxes, which act as reservoirs for the parasite [2]. Over 20 species of Leishmania are known to infect humans, leading to a wide range of clinical manifestations. These forms are generally categorized into three distinct types: visceral leishmaniasis (VL), cutaneous leishmaniasis (CL), and mucocutaneous leishmaniasis (MCL) [2,3].

The World Health Organization (WHO) classifies leishmaniasis as a neglected tropical disease (NTD). It is particularly common among populations with limited socio-economic resources, where unfavorable conditions promote the proliferation of vectors [3,4,5]. According to the WHO Global Health Observatory [6,7], leishmaniasis is endemic in 98 countries and territories, affecting approximately 350 million people and accounting for 12 million infections. The estimated annual incidence ranges from 50,000 to 90,000 VL cases and from 600,000 to 1 million CL cases, with an overall estimate of 700,000 to 1 million new cases each year [1,7]. In South America, Brazil and Colombia exhibit the highest incidence rates of cutaneous leishmaniasis (CL), while visceral leishmaniasis (VL) is reported less frequently than in other regions worldwide [8,9,10].

The activity, survival, and population density of vectors and reservoirs can be influenced by various factors, including temperature, humidity, rainfall, frost line, soil types, and other climatic conditions. These variables determine the availability of suitable mammalian hosts (reservoirs) and Lutzomyia species (vectors), which in turn define the incidence of leishmaniasis in a specific region as well as the clinical manifestations and epidemiological patterns [11]. In Ecuador, the most prevalent *Leishmania* species are *Leishmania (Viannia) braziliensis*, *Leishmania (Viannia) guyanensis*, and *Leishmania (Leishmania) amazonensis*. *L. braziliensis* and *L. panamensis* are frequently identified in Andean regions and are associated with LC in [11,12].

**Figure 1:**
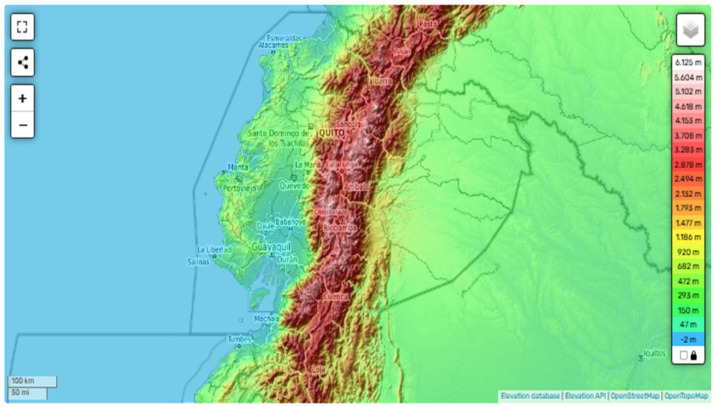
Topographic map of Ecuador showing the three continental regions—Coast (0–500 m, higher temperatures), Andes (500–6126 m; lower temperatures), and Amazonia (200–1000 m; higher temperatures)—highlighting elevation gradients and associated climatic zones. From: OpenStreetMap contributors. Topographic map of Ecuador [Internet]. OpenStreetMap; n.d. [accessed on 1 September 2025]. Available at: https://es-ec.topographic-map.com/map-m3ggp/Ecuador/?center=-1.41709%2C-78.32703&zoom=7&base=2 [16].

Ecuador is a country in the Andean region of South America, bordered by Colombia and Peru and divided into four natural regions. The continental regions cover an area that stretches from the coast of the Pacific Ocean to the Amazon Forest. They are characterized by tropical weather and high humidity, with altitudes below 1000 m above sea level (m.a.s.l.), known as the lowlands. The highland region, which includes the Andes and their slopes, runs through the country from north to south, separating the coastal and Amazon regions (Figure 1) and creating diverse landscapes with lower temperatures and tall mountains, reaching altitudes of up to 6000 m.a.s.l. [13]. Typically, the Andean region is characterized by two well-defined seasons profoundly influenced by the El Niño-Southern Oscillation (ENSO) phenomenon: a dry season from March/April to November/December with few clouds, higher evaporation rates, and a completely dry ground and a rainy season from January to March with abundant rains and permanently cloudy skies [14,15].

Historically, leishmaniasis in Ecuador has been associated with humid tropical and subtropical climates, particularly in rural areas of the Coast and Amazon regions. However, recent surveillance data from the National Directorate of Epidemiological Surveillance (SE01-52) revealed a notable shift: in 2019 and 2020, Pichincha Province—located in the Andean region—ranked second in the number of reported cases. While Morona Santiago in the Amazon region reported the highest incidence, as expected due to its geographic and socioeconomic characteristics, Pichincha’s case numbers exceeded those of several provinces in the Coast and Amazon regions with similar environmental conditions. CL was by far more prevalent than MCL in all the reporting regions [17].

**Figure 2:**
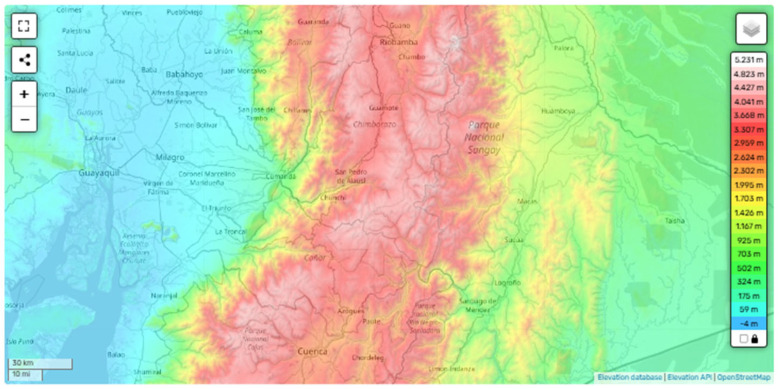
Topographic map of the central Andean region of Ecuador, including the province of Chimborazo. The map displays elevation gradients ranging from 2376 m to 5041 m.a.s.l., using a color scale from green (lower elevations, higher temperatures) to red (higher elevations, lower temperatures). The region is characterized by an equatorial high-mountain climate, with average temperatures around 8 °C and frequent drops below 0 °C at higher altitudes. These environmental conditions, namely low temperature, reduced humidity, and high elevation, are generally unsuitable for the survival of Lutzomyia sandflies and the transmission of Leishmania parasites. Despite this, confirmed cases of leishmaniasis have been reported in several cantons, suggesting a possible shift in ecological suitability due to climate change. From: OpenStreetMap contributors. Topographic map of the Province of Chimborazo [Internet]. OpenStreetMap; n.d. [accessed on 1 September 2025]. Available at: https://es-ec.topographic-map.com/map-xg457/Cuenca/?center=-2.27513%2C-78.68716&zoom=9 [16].

This data is noteworthy, as the climate conditions of the Andes are inconsistent with the typical environments in which leishmania vectors typically thrive. The province of Pichincha, located in the northern Andes of Ecuador, exhibits a diverse topography ranging from high-altitude peaks exceeding 4700 m to inter-Andean valleys situated around 2400 m.a.s.l. The region experiences a subtropical highland climate, with average temperatures varying between 12 °C and 25 °C depending on elevation and season. The landscape includes mountainous terrains, river-irrigated valleys, and densely populated agricultural zones. These environmental conditions, combined with moderate humidity and seasonal rainfall, create microclimates that may support the survival of leishmania vectors, particularly in lower-altitude valleys surrounding the capital city, Quito [13,18]. In 2020, the province of Chimborazo, located in the central Andean highlands of Ecuador, reported cases of leishmaniasis despite its traditionally unfavorable environmental conditions for vector survival. With a median altitude of approximately 3900 m above sea level and an equatorial high-mountain climate, the region experiences average temperatures around 8 °C, with minimums often dropping below 0 °C and maximums rarely exceeding 20 °C. These climatic characteristics have historically limited the presence of Lutzomyia sandflies, making the emergence of leishmaniasis in this area unexpected (Figure 2) [17,19].

Climate projections using the PRECIS model (Marengo et al., 2009) indicate that by 2071–2100, tropical South America will experience more frequent warm nights, fewer cold nights, and intensified rainfall events [20]. In line with these forecasts, Chimborazo Province, which has undergone significant deforestation, is projected to experience a rise in average monthly temperatures by 2050, consistent with regional climate change models and suggesting a shift toward more favorable conditions for vector-borne diseases, which due to their potential epidemiological implications warrant further investigation [21].

## 2. Materials and Methods

### 2.1. Ethics Statement

The study was conducted with authorization from the Faculty of Medicine Academic Unit at the Pontificia Universidad Católica del Ecuador and clearance from the Human Research Ethics Committee (code EO-212-2022, V1, date 4 January 2023). This was done to ensure that the research adhered to bioethical standards. The investigation utilized data collected from hospitals and health centers, excluding patient-identifying information and thus eliminating the need for informed consent.

### 2.2. Study Design

This study employed an observational ecological design based on secondary data obtained from the District Directorate of Zone 3 of the Ecuadorian Ministry of Public Health (MSP), under the Health Coordination of Chimborazo Province. The analysis focused on confirmed cases of leishmaniasis reported between 2013 and 2022 in the cantons of Alausí and Chunchi. The ecological approach allowed us to explore associations between environmental variables—such as temperature, humidity, and altitude—and the spatial and temporal distribution of leishmaniasis cases. By integrating epidemiological records with regional climate data, the study aimed to identify patterns that may reflect the influence of climate change on disease transmission in high-altitude Andean settings.

### 2.3. Overview of the Geographical Area Under Investigation

The study focused on specific areas of Chimborazo Province, which covers approximately 6499.72 km^2^ and includes high-altitude zones such as the Chimborazo Volcano (6310 m.a.s.l.). The province experiences distinct wet and dry seasons, with the wettest months occurring from January to April. Temperatures typically range from 4 °C to 15 °C year-round, although the valleys of the province may differ in this regard [17,19,20].

The two primary locations of our study are the valleys of Alausí and Chunchi, in the southern part of Chimborazo Province. At an elevation of 2428 m, Alausí and Chunchi experience temperatures ranging from 6 °C to 16 °C and receive an annual rainfall of 406 mm. Huigra, a parish within Alausí and located at 1219 m, has a subtropical climate characterized by average temperatures between 18 °C and 19 °C and an annual rainfall of 474 mm. These inter-Andean valleys may create microclimates that are more conducive to vector survival [22].

### 2.4. Sample and Setting

In the observational study, data were collected from the Ecuadorian Health Care Records Platform (PRAS) for the cantons of Alausí and Chunchi. The inclusion criteria consisted of (1) a confirmed diagnosis of leishmaniasis, established through either parasitological or serological testing conducted in Ministry of Health (MSP)-approved laboratories; (2) individuals seeking medical care at hospitals or health centers within these cantons between 2013 and 2022; (3) permanent residents who have always lived, currently reside, and work in these areas, excluding mobile populations; and (4) accessible PRAS records. The exclusion criteria included (1) cases without laboratory confirmation; (2) duplicate or incomplete records lacking essential demographic or diagnostic information; and (3) patients living outside the specified geographic areas. Diagnostic tests were performed in MSP-approved laboratories, adhering to established quality control standards that ensure high specificity and sensitivity. The results are considered reliable based on positive predictive values. The analysis focused solely on confirmed cases, regardless of the causes or clinical symptoms.

### 2.5. Data Analysis

Statistical analysis was performed using SPSS software (version 25), where we applied the χ^2^ and Cramer’s V tests to assess the categorical variables (*p* = 0.05). Two-sided exact binomial tests (*p* = 0.5) were used to evaluate differences in sex and marital status distributions among leishmaniasis cases. The Quantum Geographic Information System version 3.30.2 (QGIS) was utilized to visualize and analyze the distribution of reported leishmania cases by integrating the disease data into the geographic information system. Additionally, non-parametric Spearman’s rank correlation analyses were conducted to explore associations between environmental variables and disease prevalence during the peak years of 2018 and 2019.

## 3. Results

### 3.1. Annual Distribution of Leishmaniasis Cases in the Cantons of Alausi and Chunchi and Their Respective Parishes from 2013 to 2022

**Figure 3:**
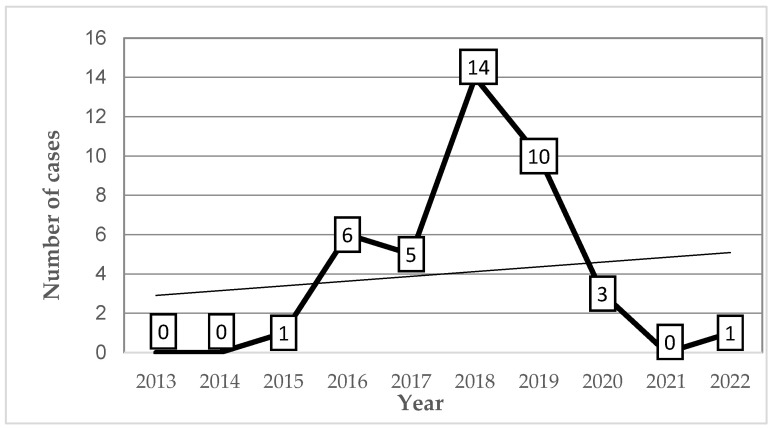
Annual distribution of leishmaniasis cases in the cantons Alausi and Chunchi, 2013–2022. The figure displays the number of reported leishmaniasis cases per year in the canton of Alausi that includes the city of Alausi and the parish of Huigra, the two cities where the cases were reported. A notable increase occurred between 2016 and 2019, peaking in 2018 with 14 cases. The trend declined thereafter, with only one case reported in 2022. No cases were recorded in 2013, 2014, and 2021.

Between 2013 and 2022, a total of 40 leishmaniasis cases were documented in the province of Chimborazo, with a mean annual incidence of 1.04 cases per 100,000 population. The data reveal a fluctuating trend, with a modest increase in cases between 2015 and 2017, followed by a sharp rise in 2018, reaching a peak incidence of 3.51 per 100,000 and 24 cases reported between 2018 and 2019. After this peak, a consistent decline was observed, culminating in just four cases and an incidence of 0.26 per 100,000 in 2022. Notably, no fatalities or hospitalizations related to leishmaniasis were recorded during the study period (Figure 3).

### 3.2. Monthly Distribution of Leishmaniasis Cases in the Cantons of Alausi and Chunchi

**Figure 4:**
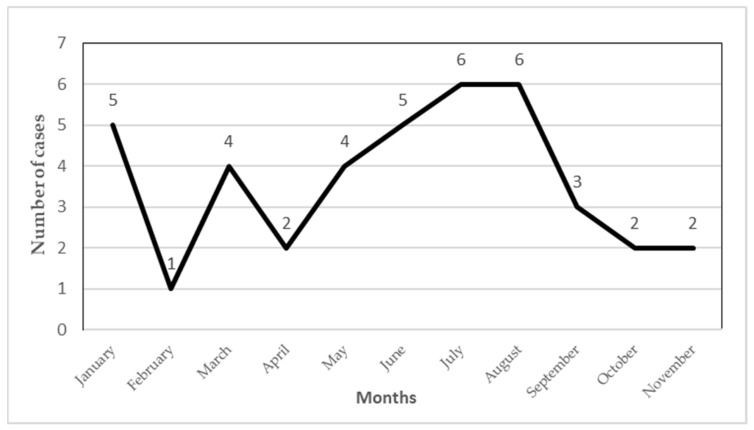
Monthly distribution of leishmaniasis cases in the cantons of Alausi and Chunchi from 2013 to 2022.

The monthly trend of leishmaniasis cases revealed two peaks: a significant peak in July and August, with six cases each, and a smaller peak in January, with five cases. Leishmaniasis cases gradually increased from two in April to six in August—months characterized by the so-called summer, a less rainy season—followed by a notable decrease from September to November (three to two cases) and no reports in December, the rainier and colder months of the year. Statistical analysis indicated significant differences in the monthly reports of leishmaniasis cases (*p* = 0.001). Overall, the trend in leishmaniasis cases in Chimborazo showed a fluctuating pattern, with a notable decrease towards the end of the study period. No fatalities were reported during this time. Figure 4 provides a visual representation of this data.

### 3.3. Spatial Distribution of Leishmaniasis in the Province of Chimborazo

**Figure 5:**
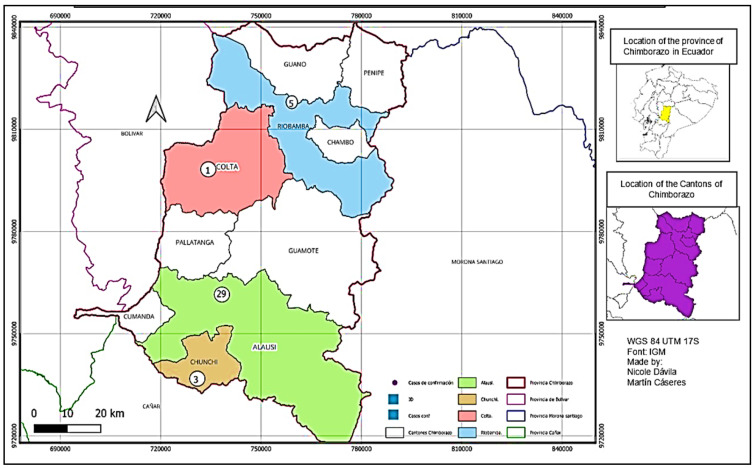
Distribution of leishmaniasis cases by canton in the province of Chimborazo from 2013 to 2022. The map highlights the cantons of Chimborazo, including Alausí, Chunchi, Riobamba, Colta, Guano, Chambo, Penipe, Cumandá, and Pallatanga. Each canton is color-coded for clarity. Insets show the location of Chimborazo within Ecuador and the relative position of the cantons within the province. This spatial representation supports the analysis of leishmaniasis case distribution in relation to altitude and climate variability across the province. The numbers shown in this figure represent cases of cutaneous leishmaniasis with the most evident clinical manifestations, which triggered public health alerts in the province due to the lack of cases reported previously.

The distribution of leishmaniasis cases in the province of Chimborazo from 2015 to 2022 varied by canton. The southern region, particularly the canton of Alausi, reported a total of 34 cases. In contrast, the canton of Riobamba, located in the northern part of the province, had five cases, while Colta reported one case, and Chunchi reported six cases. The altitudinal ranges for Colta and Riobamba are 2754 and 3212 m above sea level, respectively. Meanwhile, the altitudes for the neighboring cantons of Alausi and Chunchi are 2340 and 2548 m above sea level, respectively. No cases were reported in the other cantons, as illustrated in Figure 5.

### 3.4. Spatial Distribution of Leishmaniasis Cases in the Valley of Alausi and Chunchi and Its Parishes

A second spatial distribution analysis was conducted at the parish level in the canton of Alausí, where 34 cases of leishmaniasis were reported. Our findings indicated that the canton of Alausí accounted for 90% of the reported cases, while the canton of Chunchi reported only six cases, corresponding to 10%. The Alausí Central parish, in the center of the canton, had the highest number of cases, totaling 22 and accounting for 64.70% of the total. The Huigra parish followed with four cases, representing approximately 11.76%. Additionally, the Guasuntos parish reported three cases, constituting about 8.82% of the cases in that canton. The parishes of Simbabe, Lizarzaburu, Achupallas, Sevilla, and Nariz del Diablo each reported one case, collectively accounting for 14.7%. In contrast, the canton of Chunchi reported six cases, all originating from the Gonzol parish, representing 100% of the reported cases in that canton.

**Figure 6:**
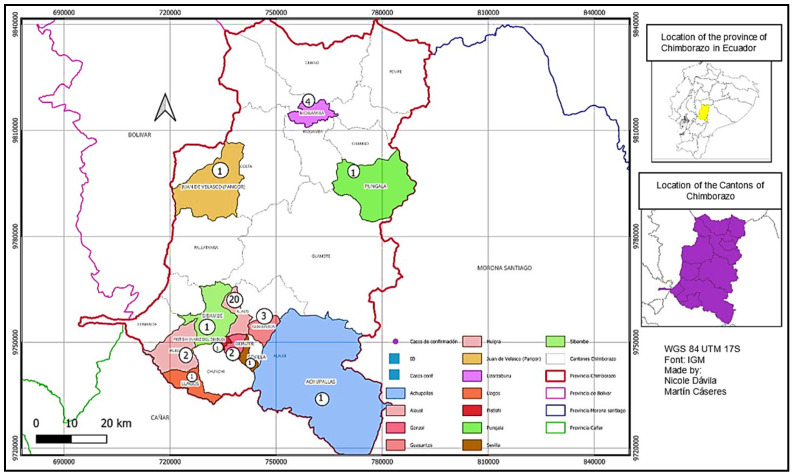
Distribution of Leishmaniasis by parish, cantons of Alausi and Chunchi, 2013–2022. This map shows the distribution of Leishmaniasis cases by parish within the cantons of Alausí and Chunchi. These two cantons drew particular attention for reporting the highest number of cases during the study period. The numbers on the map represent cases of cutaneous leishmaniasis with the most evident clinical manifestations and easy diagnosis. Color coding is used to differentiate parishes, and inset maps show the location of Chimborazo province within Ecuador and the relative position of the cantons.

The spatial distribution analysis (Figure 6) revealed that the majority of leishmaniasis cases in the canton of Alausí were concentrated in the Alausí Central parish, with only 10% of reports coming from the canton of Chunchi, which shares geographical boundaries with Alausí [23].

### 3.5. Type of Leishmaniasis

In line with previous studies on the types of leishmaniasis prevalent in Ecuador, an analysis of data from the Zonal Health Coordination 3 of Chimborazo shows that between the years 2003 and 2022, the reported cases in this province were primarily LC (*n* = 39) and LMC (*n* = 1). The reported ratio of mucocutaneous leishmaniasis to cutaneous leishmaniasis (MCL:CL) in this province (1:39) is lower than the overall ratio found across the country, which is 1:13 [24].

### 3.6. Demographic Characterization of Reported Cases of Leishmaniasis

#### 3.6.1. Distribution of Leishmaniasis Cases by Sex

Depending on the type of leishmaniasis, a predominance of the male sex (*n* = 28; 70) over the female sex (*n* = 12; 30%) was found for both LC and LMC forms. An exact binomial test against a 50:50 ratio revealed a statistically significant difference (*p* = 0.017), indicating that males are significantly more affected than females (Table S1).

#### 3.6.2. Distribution of Leishmaniasis Cases by Marital Status

The study included a total of 40 confirmed cases of leishmaniasis. Among these, 35 individuals were single (including the one mucocutaneous case), equivalent to 87.50%, and 5 (12.5%) were married. An exact binomial test showed a statistically significant difference (*p* < 0.001), suggesting that single individuals are disproportionately affected (Table S2).

#### 3.6.3. Distribution of Leishmaniasis Cases by Age

**Figure 7:**
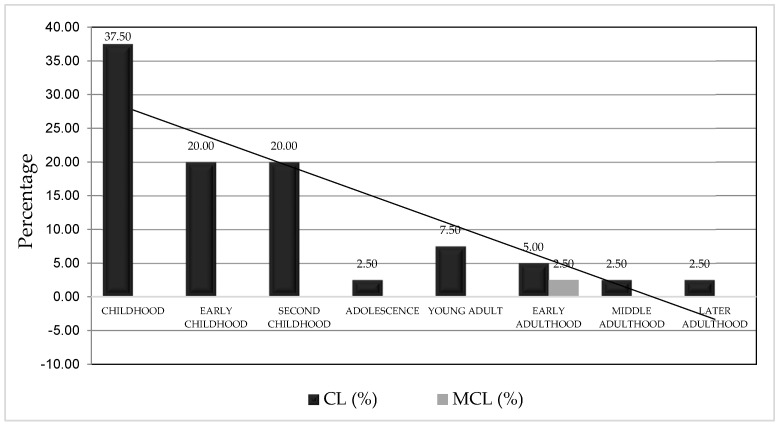
Distribution of cutaneous leishmaniasis cases by life cycle stage in the cantons of Alausí and Chunchi, Chimborazo Province, Ecuador (2013–2022). The figure illustrates the percentage of confirmed leishmaniasis cases across different age stages.

The population was categorized into different life cycle stages to understand which age groups are most affected by leishmaniasis. The findings indicate that leishmaniasis impacts all age groups. Childhood accounts for the highest percentage of cutaneous leishmaniasis (CL) cases (37.5%), with early and late childhood each accounting for 20% of cases. Chronic mucocutaneous leishmaniasis (CML) cases were reported only in early adulthood (2.5%). There were no statistically significant differences in the occurrence of leishmaniasis among the age groups based on life cycle stages (Cramer’s V = 0.56, *p* = 0.081). Figure 7 displays the distribution of leishmaniasis cases according to the life cycle stages at the time of evaluation.

#### 3.6.4. Number and Distribution of Leishmaniasis Lesions

In the past ten years, 40 reported cases of leishmaniasis were registered in the archives analyzed. However, only 19 of these reports provided information on the number of lesions in children and adults. Our analysis reveals that 85.71% (*n* = 17) of these reports indicated the presence of single lesions, while 14.29% (*n* = 2) reported multiple lesions. Regarding the lesions from leishmaniasis, they are categorized into three regions: the face, upper extremities, and lower extremities. Our study revealed that the face accounted for 88.24% (*n* = 19) of the lesions, while the upper extremities accounted for 11.76% (*n* = 2). These results align with global research indicating that leishmaniasis lesions are more frequently observed on the face than on the upper extremities.

### 3.7. Environmental Correlation Analysis of Leishmaniasis Prevalence in Alausí (2018–2019)

**Figure 8:**
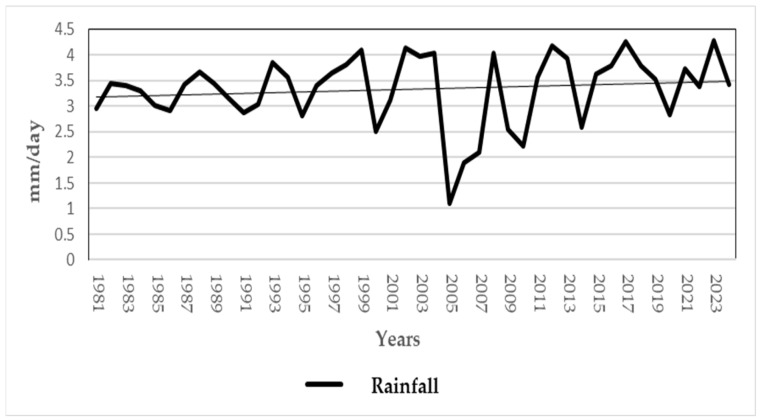
Rainfall trend in the Alausí Area, Ecuador (1981–2024). The graph displays fluctuations in precipitation (mm/day), with notable variability across the years. The years 2018 and 2019 correspond to the highest prevalence rates of mucocutaneous leishmaniasis, coinciding with cases reported in the greatest number of months. In 2018, a reduction in minimum temperature and rainfall was associated with decreased disease prevalence, with the lowest reduction observed in September. These climatic patterns may have influenced vector ecology and transmission dynamics in the region. Font: NASA.org.

**Figure 9:**
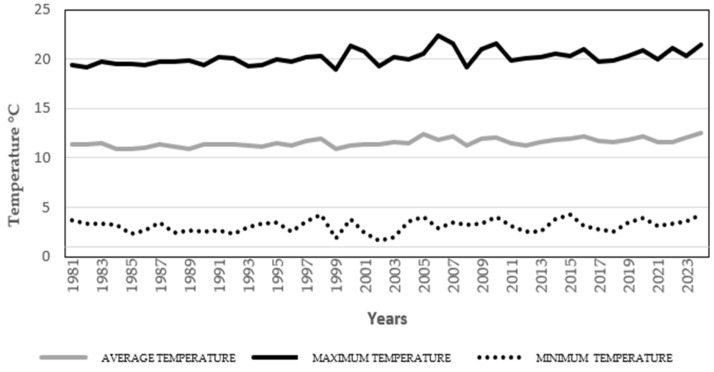
Temperature trend in the canton of Alausí, Ecuador (1981–2024). The graph displays average (gray), maximum (black), and minimum (round dots) temperatures in degrees Celsius. These long-term climatic patterns show a clear increase in the average and maximum temperature of the valley of Alausi and provide context for understanding seasonal and interannual variations in leishmaniasis transmission, particularly in relation to vector ecology and parasite development. Font: NASA.org.

**Figure 10:**
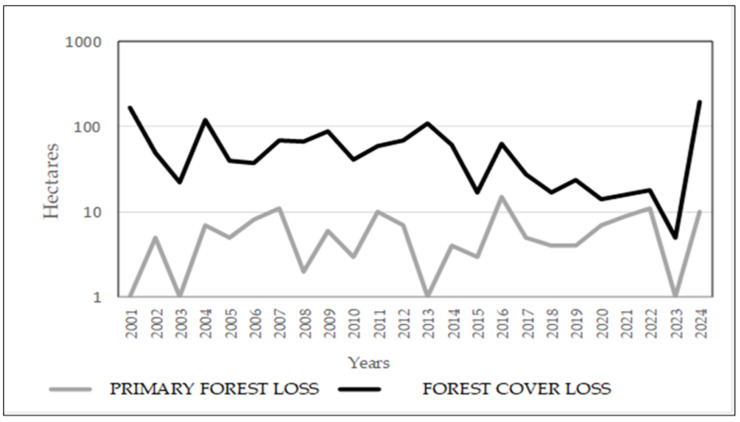
Loss of primary forest and tree cover in the Alausí area, Ecuador (2001–2024). The graph displays annual deforestation trends, with the blue line indicating loss of primary forest and the orange line representing overall tree cover loss, measured in hectares on a logarithmic scale. A continuous primary deforestation process is observed, which experienced a sharp increase in 2024, contributing to the loss of tree cover. Font: Global Forest Watch.

To contextualize the emergence of leishmaniasis in the Andean region, we examined long-term environmental trends. Figure 8 and Figure 9 present rainfall and temperature data for the Alausí area from 1981 to 2024, offering insight into seasonal and interannual variability that may influence vector ecology and parasite development. Figure 10 complements this analysis by illustrating the progressive loss of primary forest and tree cover between 2001 and 2024. Deforestation and habitat fragmentation are known to disrupt vector habitats and alter human exposure patterns, potentially contributing to changes in disease transmission dynamics.

In light of the notable increase in leishmaniasis cases during 2018, we conducted non-parametric correlation analyses to explore potential associations between environmental variables and disease prevalence. A Spearman’s rho of −0.563 (*p* = 0.057) was observed between cutaneous leishmaniasis prevalence and relative humidity and a rho of −0.562 (*p* = 0.057) with average temperature. These findings suggest a moderate inverse relationship, although it is not statistically significant at the conventional threshold (Supplemental Figure S1).

Given that 2019 also exhibited a consistent monthly distribution of mucocutaneous leishmaniasis cases, we applied the same non-parametric statistical approach used for the 2018 data. As shown in Supplemental Figure S2, April marked a distinct reduction in disease prevalence, prompting further analysis of its association with environmental variables.

The strongest correlation identified was between prevalence and average temperature, with a Spearman’s rho of −0.505 and a *p*-value of 0.094. Although this result does not reach statistical significance, it mirrors the inverse relationship observed in 2018, reinforcing the hypothesis that temperature may play a role in modulating transmission risk. These findings, while preliminary, suggest that climatic factors—particularly temperature and humidity—could influence the seasonal dynamics of leishmaniasis in the Andean region.

## 4. Discussion

### 4.1. Environmental Shifts and Epidemiological Patterns

This cross-sectional exploratory ecological study examined the morbidity and mortality profile of leishmaniasis in the Chimborazo province, an Andean region of Ecuador, during the period 2013–2022 and investigated if climate changes caused by wild deforestation, temperature increase, and changes in rainfall patterns could influence the presence of leishmaniasis in places not reported before. Our findings revealed the unexpected presence of 40 confirmed leishmaniasis cases, predominantly cutaneous (97.5%) and mucocutaneous (2.5%), in an area traditionally characterized by a cold climate and high altitudes. No fatalities were recorded. These results not only highlight a shifting epidemiological landscape for leishmaniasis in Ecuador but also demonstrate the urgent need to understand the profound implications of climate change on the distribution and incidence of this neglected tropical disease in traditionally non-endemic, high-altitude regions.

The appearance of leishmaniasis in previously unaffected areas of the Andean region raises important questions about the role of environmental change in disease dynamics. To investigate this, we conducted Spearman’s rank correlation analyses to assess potential associations between monthly disease prevalence and environmental variables during the years 2018 and 2019. We observed a moderate inverse relationship between prevalence and average temperature in both years, but the results did not reach statistical significance at conventional thresholds.

Although not statistically significant, these findings support the hypothesis that temperature and humidity may influence transmission risk, potentially by affecting sandfly activity, parasite development, or human–vector interactions. The consistency of the inverse relationship across both years suggests that lower temperatures and humidity levels may be associated with reduced transmission, warranting further investigation.

Nevertheless, these associations must be interpreted with caution due to the complex relationship between environmental conditions, vector behavior, and human exposure. Factors such as delayed healthcare-seeking behavior, long incubation periods, and reporting delays may obscure the actual timing of exposure. Additionally, deforestation and land-use changes may alter vector habitats and migration patterns, contributing to the emergence of cases in previously unaffected areas.

To better understand these dynamics, future research should incorporate more granular data on lesion onset, vector density, and microclimatic conditions. Such data would enhance predictive models and support the development of targeted surveillance and control strategies, particularly in ecologically sensitive regions undergoing rapid environmental change.

### 4.2. Leishmaniasis in Andean Regions: A Shifting Paradigm Under Climate Change

Historically, leishmaniasis in Ecuador has been predominantly associated with humid tropical and subtropical climates, particularly in the coastal and Amazonian lowlands [12,13]. The prevailing understanding has been that the environmental conditions in the Andean highlands, characterized by lower temperatures and higher altitudes, are generally unfavorable for the proliferation and survival of *Lutzomyia* sandfly vectors [5,8]. However, the detection of 40 cases in Chimborazo, a province with an average altitude of 3900 m.a.s.l. and temperatures from 0 °C to 20 °C, fundamentally challenges this long-held view. This fluctuation in the number of cases over the years suggests a potential pattern or trend that various factors may influence. Further analysis is needed to understand the reasons behind these fluctuations and develop prevention and control strategies.

Our findings are consistent with a growing body of evidence from South America that suggests an altitudinal expansion of leishmaniasis, likely driven by climate change [1,4]. While early studies on Andean cutaneous leishmaniasis (Andean CL, locally known as “uta” in Peru and Ecuador) acknowledged its presence in specific inter-Andean valleys [4,5,8], the occurrence of cases in a province like Chimborazo, with a significant proportion of its territory at high altitudes, points to a broader and more widespread phenomenon.

The projected increases in minimum and maximum temperatures for Chimborazo by 2050 [24] are particularly pertinent. These warmer conditions could create more permissive environments for Lutzomyia species, allowing them to adapt to higher altitudes and previously unsuitable thermal niches. This aligns with models that predict shifts in vector distributions due to climate change, although some models have also suggested potential declines in vector prevalence in certain areas [2]. However, our empirical data from Chimborazo indicates an expansion of endemic areas, suggesting a net increase in risk in the Andean highlands.

The observed seasonality, with peaks in leishmaniasis cases during July and August (less rainy months) and lower incidence in December (rainier and colder months), suggests a linkage to the vector’s life cycle and activity patterns, as commonly seen in other endemic regions [23]. As climate change alters precipitation patterns and temperatures, these seasonal dynamics could become more pronounced or even shift, potentially extending the transmission season in high-altitude areas. This necessitates a re-evaluation of current surveillance and control strategies, which are often designed for historical endemic zones and their established seasonal cycles.

### 4.3. Implications of Geographical and Demographic Distribution in a Climate Change Context

The spatial clustering of cases in the southern valleys of Chimborazo Province, particularly in Alausí and its parish Alausí Central, is a critical insight. The altitudinal range within these areas, from 1219 m.a.s.l. in Huigra to 2428 m.a.s.l. in Alausí and Chunchi, coupled with the varied Ecuadorian microclimates, suggests that specific environmental factors or ecological niches within these regions may be favorable to vector populations. This trend is alarming, as higher altitudes often have less public health infrastructure and access to healthcare, making populations in these newly affected areas particularly vulnerable.

The predominance of cutaneous leishmaniasis cases (39 out of 40 cases) found in our study aligns with the overall epidemiological profile of leishmaniasis in Ecuador, where CL is by far the most common clinical form, while visceral leishmaniasis is rare [24]. Previous molecular analyses in Andean regions have identified *Leishmania braziliensis* and *Leishmania panamensis* as common causative agents [12,13]. These species exhibit genetic complexity and adaptability to diverse ecological niches. Although the Andes have traditionally been considered unsuitable for vector survival due to altitude and cooler temperatures, climate variability—especially phenomena like the El Niño Southern Oscillation—has been shown to alter vector distribution patterns [14]. Historical entomological data from Panama and Colombia support the idea that anthropophilic *Lutzomyia* sandflies can expand their range under changing climatic conditions [15], suggesting that warming trends and habitat disruption may be facilitating the emergence of CL in previously unaffected highland areas. Comprehensive mapping of *Leishmania* species across altitudinal gradients remains crucial for understanding parasite–vector dynamics and informing control strategies.

The demographic analysis revealed a predominance of male cases (70%) and single individuals (87.5%) among the confirmed leishmaniasis cases in Chimborazo Province. To assess whether these distributions significantly differed from the expected 50:50 ratio, we applied two-sided exact binomial tests. The result for sex distribution (28 males out of 40 cases) was statistically significant (*p* = 0.0166), indicating that men are disproportionately affected by leishmaniasis in this region. Similarly, the marital status distribution (35 single individuals out of 40 cases) showed a highly significant deviation from the expected proportion (*p* = 0.000001), suggesting that single individuals are at notably higher risk. We also observed significant clustering in children (37.5% overall, with 20% in early childhood and 20% in late childhood), indicating specific exposure patterns. The male predominance could be linked to outdoor occupational activities (e.g., agriculture, construction, etc.) that increase exposure to sandfly bites, a pattern observed in many endemic settings. The high incidence in children is particularly concerning, as they may have less developed immune responses, prolonged exposure during play or daily activities, and limited awareness of preventive measures. This finding underscores the urgent need for targeted public health interventions, including community education, vector control, and early diagnosis and treatment, specifically tailored for pediatric populations in these emerging endemic areas.

### 4.4. Clinical and Epidemiological Characterization

The type of leishmaniasis that predominates in the study areas is CL, followed by MCL, and without records of VL. According to the World Health Organization, CL is the most prevalent, with more than 12 million people infected worldwide, which means that it is 75% greater than the other two types of leishmaniasis [15].

The distribution of CL according to life cycle stages showed that no age stage is free from contracting the pathology, and a predominance is evident in the child population until second childhood; it is likely that this is due to the lack of immunity in this age group to leishmaniasis, especially at the childhood stage. It is also important to mention that *Lutzomyia* has been described as an arthropod with a low flight range [16]. Therefore, this could explain the higher prevalence group because children at that age are closer to the ground, in the first stages of self-movement.

The analysis of leishmaniasis case distribution by sex showed that both sexes were affected by this pathology, with a higher prevalence in male patients. According to some authors [17], the sandfly has a predisposition for males due to the hormonal load that allows better parasite development [18].

Lesions are classified as single and multiple. In this study, most cases in which the number of lesions was recorded presented single lesions (90%), while the rest of the population presented multiple lesions. The location of lesions varies depending on the life cycle stage of the *Leishmania* vector. In children, the vector predominantly attacks the face and neck, whereas in women, it affects the lower extremities, and in men, the upper extremities. A study in Ecuador found that most cases reported lesions on the face, which is consistent with the results of this research [25].

### 4.5. Comparison with Global and Regional Trends and Contemporary Studies

Leishmaniasis incidence in Chimborazo Province showed notable fluctuations between 2013 and 2022, with a peak in 2018–2019. This pattern resembles the outbreak observed in Sri Lanka during the same period, where climatic anomalies—such as increased temperature and humidity—were linked to vector proliferation. Similar conditions were recorded in the cantons of Alausí and Chunchi, suggesting that climate variability may have played a key role in the rise of cases in these highland area [26].

Our findings align with recent national data from Ecuador. Costa-España et al. (2024) conducted a cross-sectional national study analyzing inpatient data on dengue, malaria, and leishmaniasis from 2015 to 2022, providing a broader context for understanding leishmaniasis trends across Ecuador [27]. When reviewing the Vector Gazette, published in 2019 by the National Directorate of Epidemiological Surveillance of the Ministry of Public Health of Ecuador, a concordance was found with the data obtained in this study; the data registry at the national level was 1104 cases, and of these 10, corresponded to the province of Chimborazo; therefore, the data recorded in the Zonal Health Coordination No. 3 and the Epidemiological Gazette at the national level was corroborated [17].

Climate change impacts on leishmaniasis distribution observed in our study are consistent with findings from other regions facing similar environmental pressures. In Iran, recent studies have demonstrated comparable patterns. Bamorovat et al. (2024) conducted a longitudinal study in southeastern Iran from 1991 to 2021 documenting the elimination trend of rural cutaneous leishmaniasis, highlighting the significant role of climate change, population displacement, and agricultural transition in disease dynamics [28]. Their findings showed that the total number of zoonotic cutaneous leishmaniasis (ZCL) cases decreased from 201 in 1991 to nil in 2021, while anthroponotic cutaneous leishmaniasis (ACL) cases decreased from 979 to 651, with temperature increases and decreased rainfall and humidity observed during this period.

Additionally, Ghatee et al. (2023) characterized geographic and climatic risk factors for cutaneous leishmaniasis in the hyperendemic focus of Bam County, southeastern Iran, showing parallels with our Andean findings regarding altitudinal and climatic influences [29]. Their study found that annual temperature, maximum annual temperature, and minimum annual temperature were effective factors in CL occurrence, with an increase of one degree Celsius increasing the chance of CL by 279%, 347%, and 237%, respectively.

The ecological interactions between vectors, hosts, and parasites in high-altitude environments, as documented in our study, have found support in recent Colombian research. Posada-López et al. (2023) examined ecological interactions of sandflies, hosts, and *Leishmania panamensis* in an endemic area of cutaneous leishmaniasis in Colombia, providing valuable insights into similar eco-epidemiological dynamics in Andean environments [30]. This research emphasized the complexity of vector–host–parasite relationships in mountainous regions, which mirrors the challenges we identified in Chimborazo. Their study identified *Nyssomyia yuilli yuilli* as the primary vector and *Psychodopygus ayrozai* as a bridging species between wild and peridomiciliary cycles, demonstrating the intricate ecological relationships that influence transmission patterns in Andean regions.

The quality of life impacts and health-seeking behavior of patients in our study region can be contextualized by recent Ecuadorian research. Bezemer et al. (2023) conducted a cross-sectional study examining the quality of life of patients with suspected cutaneous leishmaniasis in the Pacific and Amazon regions of Ecuador, providing important comparative data for understanding patient experiences across different ecological zones of the country [23]. This study also investigated *Leishmania* species distribution and clinical characteristics, offering insights that complement our demographic findings [23].

In 2018, Zeleke and collaborators carried out a retrospective study of cutaneous leishmaniasis in Ethiopia over ten years (2009–2018), developing a monthly trend during the selected period and showing a significant oscillation in the prevalence rate, indicating that the highest rate corresponded to September (63.8%), followed by January (59.7%), and the month of May (56.9%), while the lowest value (49.3%) belonged to the month of June [31]. These seasonal variations across different continents highlight the universal importance of climate factors in leishmaniasis transmission while also demonstrating regional specificities that require localized understanding and intervention strategies.

## 5. Limitations and Future Research Directions: Building Resilience in the Face of Climate Change

This study’s ecological and exploratory design, based on anonymized secondary data from healthcare records, presents certain limitations. The absence of information on the specific *Leishmania* and *Lutzomyia* species involved, the environmental determinants at a very local scale, and the precise exposure routes prevent establishing direct causal relationships between climate change and disease incidence in Chimborazo. Furthermore, reliance on passive surveillance data might lead to an underestimation of the true burden of leishmaniasis due to underreporting or misdiagnosis. The lack of fatalities observed is a positive finding, possibly reflecting effective clinical management, but it does not diminish the public health challenge posed by increased morbidity.

Moving forward, comprehensive field studies are essential to unravel the complex eco-epidemiology of leishmaniasis in the Andean highlands. Such studies should include the following:

- Active case detection to obtain a more complete picture of incidence and prevalence, building on methodologies demonstrated in recent Iranian studies [29];
- Entomological surveys to identify sandfly vectors, their population densities, activity patterns, and dispersal at different altitudes [6], incorporating lessons learned from Colombian ecological interaction studies [30];
- Molecular characterization of *Leishmania* species from human cases, vectors, and reservoirs, which will help understand the specific transmission dynamics in these new foci, following approaches used by Bezemer et al. (2023) in Pacific and Amazon regions of Ecuador [19];
- Ecological niche modeling integrating detailed climatic data, land-use changes, and socioeconomic factors to predict future risk areas and assess population vulnerability [7], incorporating climate change projections;
- Quality of life assessments and health-seeking behavior studies specific to high-altitude populations, building on frameworks established for other Ecuadorian regions.

Longitudinal studies are also needed to monitor the long-term trends of leishmaniasis incidence in relation to climate variability and change in these high-altitude regions, following the 30-year longitudinal approach successfully implemented in southeastern Iran [28]. Understanding the interplay between temperature, precipitation, land-use changes, and human activities will be critical for developing effective, evidence-based surveillance and control strategies that are adaptive to the evolving epidemiological landscape under climate change.

Integrated surveillance systems should be developed that combine epidemiological data with climate monitoring, as suggested by recent national analyses of vector-borne diseases in Ecuador [27]. This approach would enable real-time risk assessment and early warning systems for emerging leishmaniasis in previously unaffected high-altitude areas.

## 6. Conclusions

The findings of this study suggest a possible influence of climate change on the morbidity and mortality profile of leishmaniasis in the Andean region of Ecuador. While the data shows a seasonal pattern with peaks during warmer months, we acknowledge the limitation that the exact exposure routes and locations for each case are not confirmed. Therefore, the hypothesis that infections were acquired within the studied region and that climate change contributed to the observed epidemiological trends must be interpreted with caution and warrants further investigation supported by more precise exposure data.

The observed peak in January, which does not align with the warmest months, may be influenced by factors such as delayed healthcare-seeking behavior, long and variable incubation periods, and reporting delays. In remote and rural areas, patients often present weeks or months after lesion onset, which complicates the correlation between diagnosis date and actual exposure period. Future studies should aim to collect more detailed data on lesion onset and exposure history to clarify these temporal associations.

Regarding the statement that “previously ‘safe’ areas are increasingly at risk”, we recognize that the data shows fluctuations rather than a consistent increase in cases. These fluctuations may reflect complex interactions between environmental, behavioral, and healthcare access factors. Further research is needed to explore these dynamics and to determine whether climate variability is contributing to changes in transmission patterns.

In conclusion, while our findings point to a potential link between climate conditions and leishmaniasis transmission, this relationship remains hypothetical and should be explored through longitudinal studies with more granular exposure and environmental data. Strengthening surveillance systems, improving healthcare access, and implementing culturally appropriate vector control strategies remain essential components of a comprehensive response to leishmaniasis in the Andean region. A One Health approach that integrates environmental, human, and animal health perspectives will be critical in addressing the evolving challenges posed by vector-borne diseases in the context of climate change.

## Data Availability

The original contributions presented in this study are included in the article and Supplementary Materials.

## Supplementary Materials

The following supporting information can be downloaded at https://www.mdpi.com/article/10.3390/tropicalmed10090254/s1, Database with the anonymized information collected from the MSP. Table S1: Percentage distribution of leishmaniasis cases by sex. Table S2: Distribution of leishmaniasis cases by marital status. Figure S1: Monthly environmental conditions and cutaneous leishmaniasis prevalence in Alausí, Ecuador, during 2018. Figure S2: Monthly environmental conditions and mucocutaneous leishmaniasis prevalence in Alausí, Ecuador, during 2019.
